# Profiling Murchison Soluble Organic Matter for New Organic Compounds with APPI- and ESI-FT-ICR MS

**DOI:** 10.3390/life9020048

**Published:** 2019-06-06

**Authors:** Jasmine Hertzog, Hiroshi Naraoka, Philippe Schmitt-Kopplin

**Affiliations:** 1 Analytical BioGeoChemistry, Helmholtz Zentrum Muenchen, 85764 Neuherberg, Germany; 2 Analytical Food Chemistry, Technische Universität Muenchen, 85354 Freising, German; 3 Department of Earth and Planetary Sciences and Research Center for Planetary Trace Organic Compounds, Kyushu University, 8190395 Fukuoka, Japan; naraoka.hiroshi.885@m.kyushu-u.ac.jp

**Keywords:** high-resolution mass spectrometry, complementary ionization sources, organic compounds, aromatic compounds, Murchison meteorite

## Abstract

The investigation of the abundant organic matter in primitive meteorite such as carbonaceous chondrites is of major interest in the field of origin of life. In this study, the soluble organic fraction of the Murchison meteorite was analyzed by atmospheric pressure photoionization (APPI) and electrospray ionization (ESI) Fourier transform ion cyclotron resonance mass spectrometry (FT-ICR MS), in both detection modes. Such an approach ensured that we obtained an extensive description of the organic matter of the CM2 meteorite. Indeed, while in total close to 16,000 unique features were assigned, only 4% are common to all analyses, illustrating the complementarity of both the detection modes and the ionization sources. ESI FT-ICR MS analysis, in negative-ion mode, ensured to observe specifically CHOS and CHNOS species, whereas the positive-ion mode is more dedicated to the detection of CHNO and CHN species. Moreover, new organomagnesium components were observed in (+) ESI. Eventually, (+) APPI FT-ICR MS analysis was a preferred method for the detection of less polar or nonpolar species such as polycyclic aromatic hydrocarbons but also heteroatom aromatic species composing the organic matter of Murchison.

## 1. Introduction

Carbonaceous chondrites are an important subset of meteorites. Among this class of meteorite, primitive objects of the solar system can be described with chemical composition comparable to those of the sun. Indeed, like the sun, volatile elements such as C, H, N, O, and S are particularly abundant in this material [1,2]. Organic chondrites are particularly of major interest due to their high-carbon content (up to several %) present in the form of complex solvent soluble and insoluble organic matter. Having a molecular insights into the meteoritic soluble organic matter is sure to increase our understanding and knowledge on the chemical evolution of organic compounds and also of biogenic elements in the light or emergence of life.

According to the petrographic type and elemental and isotopic compositions, different sub-classes can be distinguished among the carbonaceous chondrites, (i.e., CI, CM, CK, CV, CB, and CR). Within the CM2 Mighei-type, one of the most known and studied is the Murchison meteorite that fell in 1969, in Australia. Its organic chemistry has been extensively studied by performing some solvent extraction of the soluble fraction of the meteorite. This latter process is done by simply crushing the material with single or several solvents of different polarity [3].

Different techniques can be employed to analyze this complex soluble carbonaceous matter. It is possible to distinguish (i) targeted analysis of organic compounds such as with mass spectrometry coupled to gas or liquid chromatography [4,5,6,7] and (ii) non-targeted analysis such as with nuclear magnetic resonance [8,9,10]. The targeted study of the soluble organic matter of the Murchison enables to differentiate several biogenic chemical classes that have been observed such as amino acids, amides, amines, carboxylic acids, carbonyl compounds, sulfonic and phosphonic acids, hydrocarbons, alcohols, and nitrogen heterocycles [11,12,13].

Besides these techniques, the non-targeted analysis, using high-resolution mass spectrometer, was applied for the characterization of the organic compounds in meteorites. Several studies have been carried out to date on the Murchison meteorite, either on surface or on internal fragment analysis. All the performed studies involve electrospray ionization (ESI) or desorption electrospray ionization (DESI) source in both negative- and positive-ion modes. This ionization source is known to be appropriate for the ionization of medium to polar species over a wide mass range. The less polar and oxygen depleted species are not necessarily detected by this method and thus the chemical description of the material cannot be the most exhaustive. Some ionization sources can be employed for such compounds chemistry, such as the atmospheric pressure photoionization (APPI), the atmospheric pressure chemical ionization (APCI) or the laser desorption ionization (LDI). It has been already demonstrated that these techniques are complementary to ESI and ensure an exhaustive chemical descriptions of complex samples [14,15,16,17]. 

The goal of the present study is to expend the chemical description of the soluble organics of Murchison meteorite by non-targeted approach using an atmospheric pressure photoionization source (APPI) coupled to a high resolution Fourier transform ion cyclotron resonance mass spectrometer (FT-ICR MS). This method is well known to ionize the less polar to nonpolar species such as the polycyclic aromatic hydrocarbons (PAH) or unsaturated heteroatom compounds, especially in petroleum oil [18,19]. Then, the contribution of APPI to a better understanding of the chemical composition of the meteorite will be discussed by comparing the elemental compositions achieved by positive- and negative-ion ESI FT-ICR MS. 

## 2. Materials and Methods

Fresh fragment (15–30 mg) of the Murchison meteorite was first washed with analytical grade methanol prior extraction by crushing the sample in an agate mortar with 1 mL methanol. For APPI experiments, 1 mL methanol and 40 µL toluene were used for the destructive extraction. The achieved mixture was recovered in an Eppendorf vial and centrifugated. The supernatant was directly infused in the FT-ICR MS. A similar procedure was previously used in different studies [3,20].

The measurements were carried out with a 12 T FT-ICR mass spectrometer Solarix (Bruker Daltonics) and the parameters were optimized via software FTMS-Control V2.2.0 (Bruker Daltonics). For all the experiments, the mass spectra were acquired with a 4 megaword time-domain. Prior acquisition, the mass spectrometer was externally calibrated with arginine clusters (10 mg/L in methanol). 

### 2.1. ESI Analyses

The ESI source (Apollo II, Bruker Daltonics) was used in both negative- and positive-ion modes. The methanolic solutions were infused with a flow rate of 120 µL/h. The drying gas temperature and the flow rate were kept at 180 °C and 4 L/min, respectively, and the pressure of the nebulizer gas was 2.2 bar. In negative detection mode, the capillary voltage was set at 4 kV and while, in positive-ion mode, it was maintained at 3.6 kV. The negative-ion mass spectrum ranges from *m*/*z* 147 to 2000 and results from the accumulation of 5000 scans. For positive-ion mode experiment, 3000 scans were accumulated and the mass spectrum ranges from *m*/*z* 92 to 1500. 

### 2.2. APPI Analysis

The APPI source (APPI II, Bruker) was used to perform the acquisition in positive detection mode. The sample was infused with a flow rate of 900 µL/h. The drying gas temperature and the flow rate were kept at 200 °C and 2 L/min, respectively, and the pressure of the nebulizer gas was 2.5 bar. The capillary voltage was 3.5 kV and the source was heated at 350 °C. The mass spectrum was acquired between *m*/*z* 92 and 1200 and 300 scans were accumulated. 

### 2.3. Data Treatment

The achieved data were analyzed using Data Analysis 5.0 (Bruker Daltonics). The FT-ICR mass spectra were internally calibrated by using reference mass lists of known components (fatty acids) for which the mass accuracy values were lower than 200 ppb, for (−) ESI and (+) APPI, and 500 ppb for (+) ESI. The mass spectra were exported to peak lists at a signal-to-noise ratio ≥ 2. Satellite peaks and signal related to magnetron signals were removed according to the algorithm developed by Kanawati et al. [21]. Assignment of the resulting peak lists was performed by using an in-house software, Netcalc [22]. The elemental formulae were attributed within a mass accuracy window of ± 0.1, 0.2, and 0.8 ppm for the analyses performed in (+) APPI, (−) ESI, and (+) ESI, respectively. C, H, N, O, and S elements were used for assignments. In addition, Mg was also used to assign features obtained in ESI in both detection modes whereas sodium adducts were considered in (+) ESI measurements. 

Thus, thousands of features were assigned and CH, CHO, CHOS, CHN, CHON, CHONS, and CHOMg compound classes were evidenced and gathered in the Table 1.

## 3. Results and Discussion

### 3.1. (−) ESI FT-ICR MS Analysis

The description of the chemical composition of the soluble organic matter of the Murchison meteorite by (−) ESI FT-ICR MS was already studied by Schmitt-Kopplin et al. [3,20] and Hertkorn et al. [8]. Therefore, only the most relevant information will be given in this section. 

In our analysis close to 11600 features were assigned from the analysis of the Murchison in (−) ESI FT-ICR MS. As demonstrated in the Table 1, CHONS, CHON, CHOS, and CHO compound classes are majority and correspond to 31.3%, 30.7%, 22%, and 13.3% of the total assigned features, respectively. This distribution is very close to those achieved by Hertkorn et al. [8] who performed the analysis of 5 methanolic extracts of the Murchison meteorite by (−) ESI FT ICR MS. Furthermore, they calculated the H/C, O/C, N/C, and S/C ratios of the obtained raw formulae and the achieved values are similar to those calculated in this study. 

**Figure 1:**
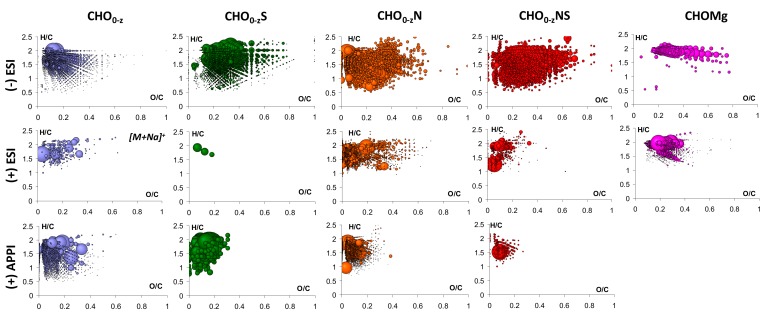
van Krevelen diagrams of the different heteroatom classes identified in the organic extract of Murchison by ESI and APPI FT-ICR MS in positive- and negative-ion modes. The bubble size refers to the corresponding signal intensity.

For each compound class, van Krevelen diagrams represented the H/C vs. O/C (Figure 1) or H/C *vs. m*/*z* (Supplementary Figure S1) were generated. 

The O/C values calculated for the CHO and CHON species extend from 0 to 0.6, whereas for the CHOS and CHONS ones, they range between 0 and 0.8. This observation suggests that the sulfur of the CHOS and CHONS species is highly oxidized, which is in agreement with previous studies [3]. Concerning the H/C values, more unsaturated species are observed within the CHOS, CHON, and CHONS classes. 

In addition to these heteroatom classes, CHOMg species were also assigned, to a lesser extent. These organomagnesium species were evidenced in slightly to heavily shocked (high pressure and temperature) meteorites as described in the work of Ruf et al. [20]. The Figure 1 shows that these species are essentially saturated (H/C = 2) and have O/C extending from 0.2 to 0.8. Moreover, these species contain between 2 and 13 oxygen atoms and cover a mass range between 200 to 650 Da. Similar observations were made by Ruf et al. [20].

### 3.2. (+) ESI FT-ICR MS Analysis

The analysis performed in ESI FT-ICR MS in positive-ion mode ensured to assign 4459 features (Table 1). Most of the assignments is related to CHON and CHN classes (64%). In addition, it is noteworthy that 786 features were related to CHOMg species. The other highlighted classes are related to CHONa and CHONS, which represent 6.4 and 11.8% of the total assigned features. Regarding CHO species, most of them are detected in the form of Na^+^ adducts. 

The assigned CHOMg species contain between and 2 and 12 oxygen atoms. According to the corresponding van Krevelen diagram (Figure 1), some of the CHOMg assignments obtained in (+) ESI are likely to be similar as some achieved in (−) ESI. These species have the following characteristics: H/C = 2; 0.2 < O/C < 0.4; and 250 ≤ *m*/*z* ≤ 650 (Figure 1 and Supplementary Figure S1). The other CHOMg assignments specifically detected in (+) ESI present a lower saturation degree (1.2 ≤ H/C ≤ 1.8). In the study performed by Ruf et al. [20], structure was suggested that can explained their detection in negative form in the form of carboxylate. In (+) ESI, some MS/MS experiments were performed on abundant CHOMg-assigned peaks to determine whether or not these species were detected in the form of [M+Mg]^+^ adducts. First, the CHOMg assignment was checked with the simulated isotopic pattern that matches (Supplementary Figure S2). A first peak at *m*/*z* 329.217405, which was assigned as C_16_H_33_MgO_5_^+^, was isolated. Mass spectra of the isolated feature, without and with (18 V) collision energy, were acquired (Supplementary Figure S3) to highlight peaks directly originated from fragmentation. For each peak resulting from collision-induced dissociation (CID), a unique raw formula was attributed. Thus, it appears that there is no loss of a magnesium cation (Mg^2+^) but only small organic moieties are removed from the parent ion leading to the detection of fragmented CHOMg cations. This indicates that magnesium is covalently bounded to the organic part. A second MS/MS experiment was carried out on a signal measured at *m*/*z* 357.212356 and assigned as C_17_H_33_MgO_6_^+^ (Supplementary Figure S4). As with the previous compound, the neutral losses correspond to small moieties, such as CO and H_2_O, and secondary CHOMg cations were still detected, demonstrating the covalent nature of the magnesium involved in these compounds. 

The CHON and CHN compounds extend between *m*/*z* 150 and 750 (Supplementary Figure S1). If more CHN and CHNO assignments were achieved in negative-ion mode than in positive-ion mode, the percentage related to these compound classes is more important in (+) ESI than (−) ESI. Generally, more high-mass CHN/CHNO compounds were observed, as illustrated on the Supplementary Figure S1 and by the higher value of the weighted average mass (Table 1). In addition, more low-oxygenated and non-oxygenated species are detected in these conditions, as demonstrated on the van Krevelen diagram (Figure 1) and by the lower value of the weighted average for the O/C. The assigned CHN species contains between 1 and 7 nitrogen atoms whereas the CHON ones contain from 1 to 12 nitrogen atoms and between 1 and 14 oxygen atoms. 

The predominance of the CHON species, and more particularly of the CHN ones, specifically detected in ESI (+), was also observed by Naraoka et al. [23,24,25,26]. The authors carried out several studies in positive-ion mode, by liquid chromatography/Orbitrap-MS or by desorption electrospray ionization/Orbitrap-MS, on the methanolic extract of the surface or on the interior Murchison meteorite. If some C_n_H_2n_NO species were detected, especially at the meteorite surface, most of the assigned species are related to CHN and CHN_2_ classes. Minor CHN_3–5_ compound classes were also identified. All these N-containing species were evidenced to be homologues of saturated and unsaturated alkylpyridines and alkylimidazoles. 

**Figure 2:**
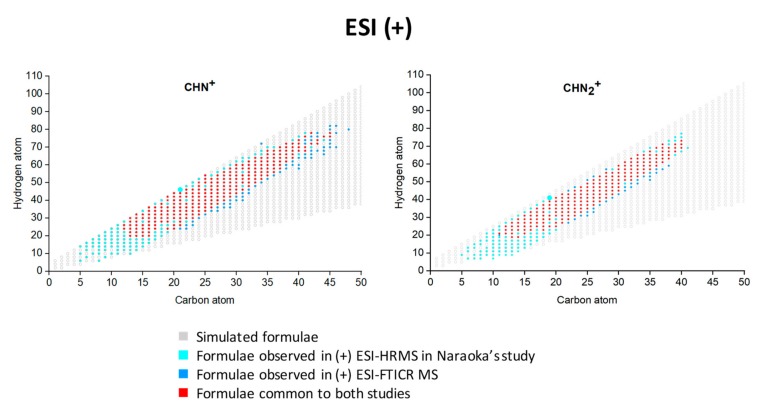
Simulated and experimental CHN^+^ formulae plotted according to their hydrogen and carbon atoms. The experimental formulae were achieved in this study in positive-ion ESI FT-ICR MS and by Naraoka et al. [24].

As part of this study, simulated CHN^+^_1–2_ species and their oxygenated (O_1_ and O_2_) homologues were generated with carbon atoms ranging from 1 to 70 and hydrogen atoms between 2 and 144. Only the raw formulae fulfilling H/C ≥ 0.75 were kept and are plotted according to their compound classes visible on Figure 2. On the same graphs, the CHN and CHN_2_ assignments obtained both here by (+) ESI FT-ICR MS and by Naraoka et al. by (+) ESI HPLC-LTQ-Orbitrap-MS [24] were superimposed. Thus, it appears that species specifically observed by Naraoka et al. are mostly lower in mass, whereas the species specifically detected in this study, by FT-ICR MS, are heavier but, more importantly, present more unsaturations. As shown on the Figure 2, some components are observed by both techniques. Indeed, close to 75% of the CHN compounds evidenced by Naraoka et al. were also observed in this study (251/335) whereas, for the CHN_2_ components, 68% are common to both analyses (190/279). This suggests that the N-containing species detected by (+) ESI FT-ICR MS are also saturated and unsaturated alkylpyridine/alkylimidazole homologues.

In addition to unsaturated CHN and CHNO species, more saturated and oxygenated compounds were specifically observed in this study. These latter components can be related to amines and amino acids/small peptides. Indeed, such compounds were evidenced in several studies performed on the Murchison meteorite [27,28,29,30]. 

The same graphical representations as those achieved with CHN compounds were done with the CHN_1–2_O_1–2_ formulae generated according to the previous parameters and those detected by (+) ESI FT-ICR MS in this study (Supplementary Figure S5). These assignments were also represented on van Krevelen diagrams (Supplementary Figure S6 and Supplementary Figure S7.A). Some patterns describing lines can be evidenced in Supplementary Figure S7.A. First, within a same molecular family, basic reactions are highlighted (Supplementary Figure S7.B) such as addition/loss of C, H_2_, CH_2_, and CH_4_. While between different heteroatom classes, different mass differences can also be evidenced which are represented in Figure S7.C. The latter figure demonstrates that these mass differences may correspond to small organic molecular building blocks, which were evidenced to be in space but also to have a significant impact on the prebiotic scenario [31,32]. The basic ones are HCN [33,34], C_2_H_3_N (acetonitrile) [35,36], CH_3_N (methanimine or methylenimine) [34,37], and CH_3_NO (formamide) [38,39,40,41].

### 3.3. Contribution of (+) APPI FT-ICR MS Analysis

The composition description of the sample achieved in (+) APPI FT-ICR MS shows high amount of CHON and CHO features (Table 1). In addition, these analytical conditions ensure to detect the highest amount of pure hydrocarbons species in regards to the ESI FT-ICR MS, in both the detection modes. CHN species are also significantly observed with close to 10% of the total assignments. APPI is known to ionize low-polar to nonpolar species characterized by a low oxygen atom number and a low saturation degree. This is illustrated in this study by the lowest value of weighted average for O/C. 

**Figure 3:**
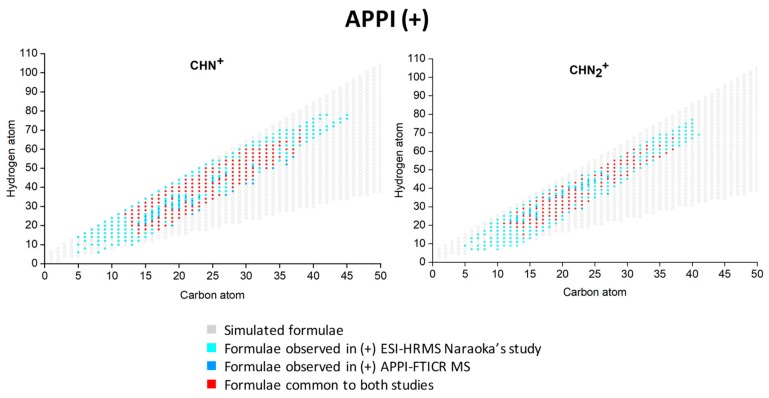
Simulated and experimental CHN^+^ formulae plotted according to their hydrogen and carbon atoms. The experimental formulae were achieved in this study in positive-ion APPI FT-ICR MS and by Naraoka et al. [24].

Concerning the CHN and CHON components ionized by (+) APPI, a poorer chemical diversity than in ESI, in both detection modes, was observed. In fact, only oxygen-poor (O/C ≤ 0.3) and non-oxygenated nitrogen species were detected. The CHN and CHNO species are detected in the form of protonated species [M+H]^+^ (82% and 90%, respectively) and radical cation M^●+^ (18% and 10%, respectively). The Supplementary Figure S8 shows that M^●+^ features concern more species with lower O/C and H/C ratios whereas the [M+H]^+^ ones concern the most saturated and oxygenated species. Such behavior was already observed in APPI [15,42] and can be explained by the fact that a radical cation appears to be more stabilized with a condensed aromatic core. The CHN assignments obtained by (+) APPI FT-ICR MS analysis were compared to those obtained by Naraoka et al. [24] (Figure 3). The same procedure as the one used with (+) EST FT-ICR MS was done. Thus, close to 67% of the 335 CHN raw formulae, assigned by Naraoka et al. in (+) ESI, are common to those detected by (+) APPI in this study (226/335). For the CHN_2_ assignments, 64% are common (179/279). The coverage of formulae between the two studies is less important than the one obtained with the (+) ESI. This assesses the fact that these species possess more polar specification and are therefore more detectable by ESI than by APPI. Again, species of lowest masses were not detected in this study as well as the more saturated species.

On the other hand, as shown in the Table 1, APPI ensures the detection of a significant amount of CHNO species. The representation for the CHN_1–2_O_1–2_ compound classes according to their number of hydrogen and carbon atoms (Supplementary Figure S9) evidences that these species are more unsaturated than those detected in ESI (+) (Supplementary Figure S5), as the H/C ratio is broader and concern lower values. 

But the APPI analysis evidences its specificity towards the low-polar to nonpolar species by the large amount of pure hydrocarbon (CH) assignments. Indeed, amongst the three different FT-ICR MS measurements performed on the Murchison meteorite in this study, the APPI-FT-ICR MS one gives an insight into this class of compounds with up to 400 unique features. 

The aromaticity equivalent (χ_c_) was calculated for the CH and CHO species, obtained by APPI and ESI FT-ICR MS in both detection modes, according to the following equation:

$$\chi_{c}=\;\frac{2C+N-H-2mO}{\mathrm{DBE}-mO}+1$$

where *m* is the fraction of oxygen atoms involved in the π-bound structure of the compound.

**Figure 4:**
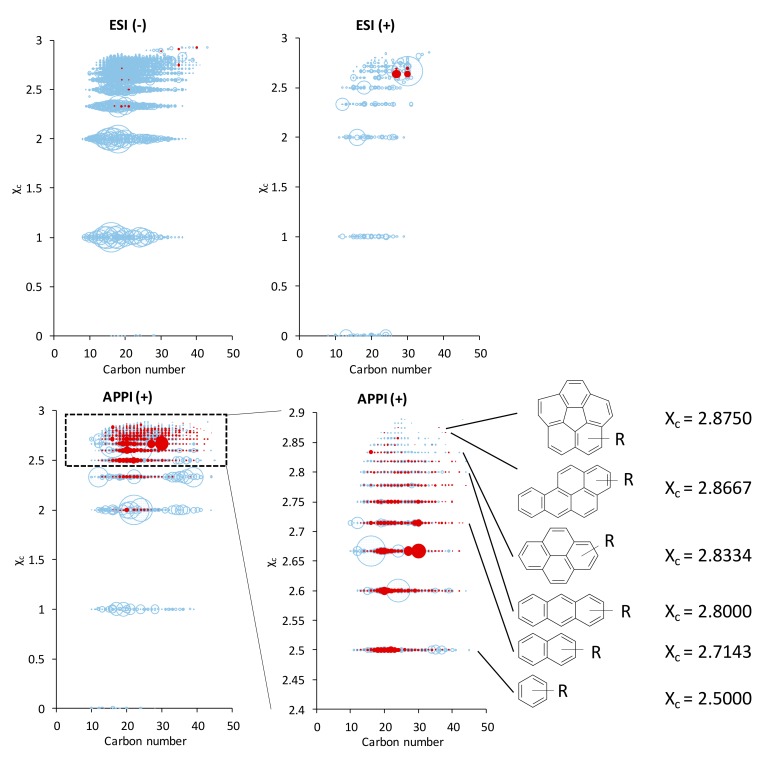
Aromaticity equivalent (χ_c_) calculated for m = 0, for CH and CHO assignments obtained by ESI and APPI FT-ICR MS, in both detection modes. An expansion was done on the most unsaturated species detected in (+) APPI FT-ICR MS. CHO specie are in blue and CH species are in red.

According to the nature of the chemical functions, *m* can be equal to 0, 0.5, and 1, as detailed in the study elsewhere [43]. Figure 4 and Supplementary Figure S10 show the calculated values of χ_c_ for the CH and CHO species according to the carbon atom number. Both the global graph and its enlargement demonstrate that a significant amount of the pure hydrocarbons detected in (+) APPI are aromatics.

Some of the CH raw formulae achieved by APPI are in agreement with some species identified by GC-MS in Murchison. Among the pure hydrocarbons components composing the Murchison meteorite, aromatics and polycyclic aromatic hydrocarbons (PAH) are subject to studies [5,7,44,45,46]. Thus, Krishnamurthy et al. [46] identified several PAH by GC-MS and some of the achieved raw formulae and, therefore, species, are common to the CH assignments obtained in this study by (+) APPI FT-ICR MS. Among the achieved raw formulae, some of them can be putatively assigned as phenanthrene, pyrene, and chrysene derivatives, as well as fluoranthene. Additional PAH species putatively assigned are given in Table 2. These species are mainly detected in the form of [M+H]^+^ and, to a lesser extent, M^●+^.

The other class of pure hydrocarbons composing the Murchison meteorite are the aliphatic ones. In different studies, branched alkyl-substituted mono-, di-, and tricyclic alkanes were evidenced in Murchison by GC-MS [46,47]. Such species have their aromaticity equivalent comprised between 0 (for an alkane) and 2.3334 (for a tricyclic alkane such as adamantane). Some of the detected species share similar characteristics as the number of C and H atoms, and χ_c_ values. 

In addition to PAH, some CHO heteroatom aromatic compounds were detected. These species have χ_c_ values equal or greater than 2.5, and some of the obtained raw formulae were common to those of some molecules identified by Krishnamurthy et al. [46] by GC-MS, as reported in the Table 2. Thus, APPI is the technique allowing detection of the highest number of CH and CHO aromatics in comparison with ESI (Figure 4). 

### 3.4. Comparison of the Achieved Data

The comparison of the assigned features obtained by (−) ESI, (+) ESI, and (+) APPI FT-ICR MS is based on the neutral raw formulae. Thus, the detected [M+Na]^+^, [M+H]^+^, [M-H]^−^, and M^+•^ species are converted into M and the duplicate formulae were removed. 

**Figure 5:**
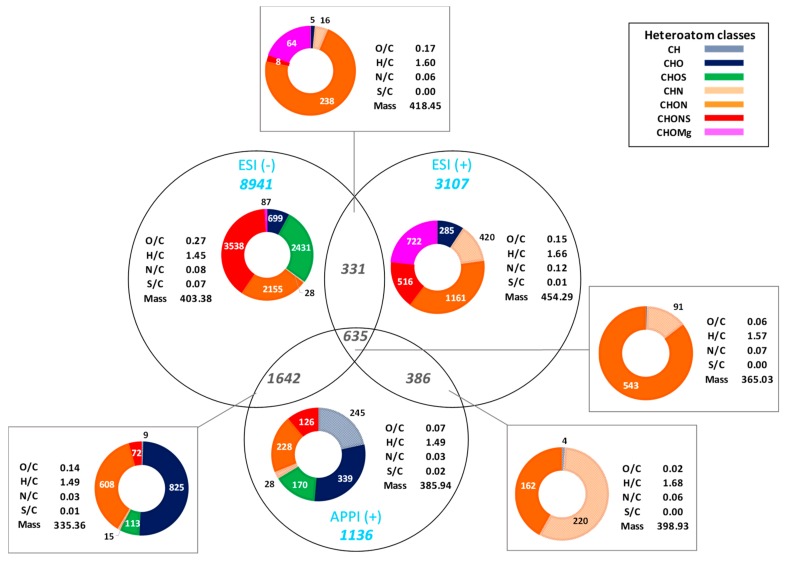
Venn diagram achieved from data obtained in ESI and APPI FT-ICR MS in both positive- and negative-ion modes. The heteroatom class distributions and the corresponding weighted average values gathered in the tables are given for specific and common features.

A Venn diagram was generated from the features obtained in APPI and ESI FT-ICR MS, in both positive and negative ion modes (Figure 5). The diagram clearly demonstrates the complementarity of all the different measurements depending on the detection mode and the ionization source. Indeed, over the 16178 assigned unique monoisotopic raw formulae, only 635 components are commonly observed in the three measurements, which corresponds to less than 4% of the total assignments. These common features are essentially species containing both oxygen and nitrogen atoms. Additional features are CHN species. 

#### 3.4.1. Contribution of the (−) ESI FT-ICR MS Analysis

From the Venn diagram, it appears that CHNOS and CHOS species are more observed in (−) ESI, this is illustrated by the highest value of $\bar{S/C}$ at 0.07. The corresponding van Krevelen diagrams (Figure 1) also show the larger chemical diversity for these classes of compounds, achieved by (−) ESI in comparison with positive-ion APPI and ESI. The sulfur involved in these components was proven to be in the form of -SO_3_ and -SH and therefore, tend to be more observed and detected in deprotonated form in negative-ion mode [3,20]. 

Moreover, the compounds specifically observed in (−) ESI tend to be more oxidized due to the highest value of $\bar{O/C}$ calculated at 0.27. Once more, the van Krevelen diagram illustrates this observation with more features plotted at higher O/C values. 

The high number of specific features (8941) obtained under these conditions shows that (−) ESI is an efficient way to ionize a significant amount of soluble species contained in carbonaceous meteorite. 

#### 3.4.2. Contribution of the (+) ESI FT-ICR MS Analysis

The (+) ESI FT-ICR MS analysis ensures the detection of additional and specific CHNO and CHN species. Indeed, most of the nitrogen-containing functions such as amines, amides, or pyridine are susceptible to be protonated and therefore detected in positive-ion mode. The achieved $\bar{N/C}$ is equal to 0.12, which represents the highest value obtained for this criterion and indicates the specificity of the (+) ESI towards these classes of compounds. 

In addition to CHN and CHNO species, this method enables the detection of additional 722 CHOMg components, which were not detected in negative-ion mode. These latter compounds are more unsaturated but less oxidized than those observed in (−) ESI. This clearly attests how important (+) ESI analysis is for the extensive description of the Murchison meteorite. 

Most of these species are detected on a greater mass range according to the weighted average of *m*/*z*.

#### 3.4.3. Contribution of the (+) APPI FT-ICR MS Analysis

The analysis of the interior fragment of the Murchison meteorite by (+) APPI analysis allows the detection of 1136 exclusive species. Although less features are observed than in ESI due to less accumulated scans, the (+) APPI is very informative in regards to pure hydrocarbon species, such as PAH and aliphatics, but also unsaturated heteroatom compounds. Due to their low polarity, these species cannot be detected by ESI, hence APPI is more suitable for their ionization. 

This class of compounds represents 90% of the carbon of carbonaceous chondrites [48] and 20% of the available carbon in the interstellar environment [49]. Regarding the prebiotic scenario, PAH are likely to play an important role. Indeed, they are considered as potential primitive pigments or building blocks for membrane cells due to their amphiphilic properties. 

## 4. Conclusions

APPI FT-ICR MS analysis of the soluble organic matter of the Murchison meteorite was carried out for the first time. This ionization source demonstrated its complementarity to the ESI source by the detection of low polar to nonpolar species such as polycyclic aromatic hydrocarbons, aliphatics, and unsaturated heteroatom components. These latter species were only observed for now by GC-MS.

In addition to the ionization source, the complementarity of both detection modes was attested in ESI, with the specific detection of sulfur-containing species in negative-ion mode whereas nitrogen components are mostly detected in positive detection mode. Some new organomagnesium species not yet ionized and detected by (−) ESI were obtained by (+) ESI. 

Thus, the complementarity of both the ionization sources and detection modes ensures the achievement of a more exhaustive qualitative composition description of the carbonaceous matter of the meteorite. Such a description is of major interest for the prebiotic scenario and, thereby, for the origin of life. 

More improvements are needed concerning the APPI acquisitions. Indeed, a significant amount of volume, and consequently, material has to be employed in this classical setup to ensure an optimal detection. Miniaturization is on-going [50] based on chip-ESI combined with Atmospheric Pressure Laser Ionization as a promising alternative to APPI for the ionization of aromatic analytes such as PAH also to approach minimal samples volumes in the light of the close coming return mission samples of Ryugu from Hayabausa2 mission and others. 

## Figures and Tables

**Table 1:** Composition description of the organic Murchison extract achieved by electrospray ionization (ESI) and APPI FT-ICR MS in positive- and negative-ion modes with number of features, corresponding percentages, and weighted averages for different atomic ratios and mass.

Heteroatom Class	ESI (−)	ESI (+)	APPI (+)
CH	12 (0.1%)	4 (0.1%)	402 (9.2%)
CHO	1530 (13.3%)	6 (0.1%)	1368 (31.4%)
CHONa	-	285 (6.4%)	-
CHOS	2544 (22%)	3 (0.1%)	286 (6.5%)
CHN	150 (1.3%)	747 (16.8%)	421 (9.7%)
CHON	3544 (30.7%)	2104 (47.2%)	1685 (38.6%)
CHONS	3618 (31.3%)	524 (11.8%)	198 (4.5%)
CHOMg	151 (1.3%)	786 (17.6%)	-
TOTAL ASSIGNED FEATURES	11549	4459	4360
Weighted average O/C	0.20	0.13	0.08
Weighted average H/C	1.67	1.74	1.71
Weighted average N/C	0.04	0.09	0.03
Weighted average S/C	0.04	0.01	0.00
Weighted average mass	320.60	444.37	353.15

**Table 2:** Some raw formulae of polycyclic aromatic hydrocarbons (PAH) and heteroatom aromatics achieved by (+) APPI FT-ICR MS and corresponding putative compound(s).

	Raw Formula	Putative Compound(s)
CH species	C_13_H_14_	Trimethylnaphthalene
	C_14_H_12_	Methylfluorene
	C_14_H_16_	Hexahydrophenanthrene/Hexyhydroanthracene
	C_15_H_12_	Methylphenanthrene
	C_16_H_16_	Hexyahydropyrene
	C_16_H_14_	Dimethlyphenanthrene
	C_16_H_10_	Fluoranthene/Pyrene
	C_17_H_12_	Methylpyrene/Benzofluorene
	C_18_H_12_	Chrysene
	C_18_H_14_	Dimethylpyrene
	C_18_H_18_	Hexahydrochrysene
	C_19_H_14_	Methylchrysene
	C_19_H_16_	Trimethylpyrene
	C_20_H_12_	Perylene/Benzofluoranthene/Benzopyrene
CHO species	C_13_H_8_O	Fluorenone
	C_13_H_10_O	Benzophenone
	C_14_H_10_O	Anthracenone
	C_14_H_8_O_2_	Anthracenedione
	C_17_H_10_O	Benzanthrone/Benzofluorenone
	C_18_H_10_O_2_	*Benzoanthracenedione*

## Supplementary Materials

The following are available online at https://www.mdpi.com/2075-1729/9/2/48/s1, Figure S1: Representation of H/C vs. *m*/*z* achieved for the different heteroatom classes identified in the organic extract of Murchison by ESI and APPI FT-ICR MS in positive- and negative-ion modes. The bubble size refers to the corresponding signal intensity, Figure S2: Experimental (grey) and simulated (green) isotopic signals for [C_16_H_33_MgO_5_]^+^ (top) and [C_17_H_33_MgO_6_]^+^ (bottom) ions identified in the organic extract of Murchison by (+) ESI FT-ICR MS, Figure S3: Fragmentation experiments performed to assess the structure of the organomagnesium component [C_16_H_33_MgO_5_]^+^ @ *m*/*z* 329.217405 in (+) ESI FT-ICR MS. On the top, mass spectrum acquired without collision energy. Below, mass spectrum acquired after isolation and fragmentation of the parent ion. Both mass spectra were acquired after 10 scans accumulation. The Table on the right gathers the different assignments obtained after fragmentation of the [C_16_H_33_MgO_5_]^+^ ion, Figure S4: Fragmentation experiments performed to assess the structure of the organomagnesium component [C_17_H_33_MgO_6_]^+^ @ *m*/*z* 357.212230 in (+) ESI FT-ICR MS. The expansion of the fragmentation mass spectrum, on the right, shows the loss of different organic moieties, Figure S5: Simulated and experimental CHN_1–2_O_1–2_^+^ formulae plotted according to their hydrogen and carbon atoms. The experimental formulae were achieved in this study in positive-ion ESI FT-ICR MS and by Naraoka et al. [24,25], Figure S6: Simulated and experimental CHN_1–2_O_1–2_^+^ formulae plotted on van Krevelen diagram. The experimental formulae were achieved in this study in positive-ion ESI FT-ICR MS, Figure S7: Simulated CHN_1–2_O_1–2_^+^ formulae plotted on van Krevelen diagram (A). Expansions (B and C) have been done to observe the different chemical reactions within a same compound class (B) or between different classes of components (C), Figure S8: van Krevelen diagram of the CHN and CHNO species assigned in the organic extract of Murchison analyzed by (+) APPI FR-ICR MS according to the detection form (protonated or radical). Bubble size is relative to signal intensity of the mass spectrum, Figure S9: Simulated and experimental CHN_1–2_O_1–2_^+^ formulae plotted according to their hydrogen and carbon atoms. The experimental formulae were achieved in this study in positive-ion APPI FT-ICR MS and by Naraoka et al. [24,25], Figure S10: Aromaticity equivalent (χ_c_) calculated with m = 0.5 and 1, for CH and CHO assignments obtained by ESI and APPI FT-ICR MS in both detection modes.
