# Supplementary material for: Profiling Murchison Soluble Organic Matter for New Organic Compounds with APPI- and ESI-FT-ICR MS
Source: Life (Basel). 2019 Jun 6;9(2):48. doi: 10.3390/life9020048 (PMC6617137; doi:10.3390/life9020048)
Supplement: Supplementary file 1 [file life-09-00048-s001.pdf]

Supplementary materials

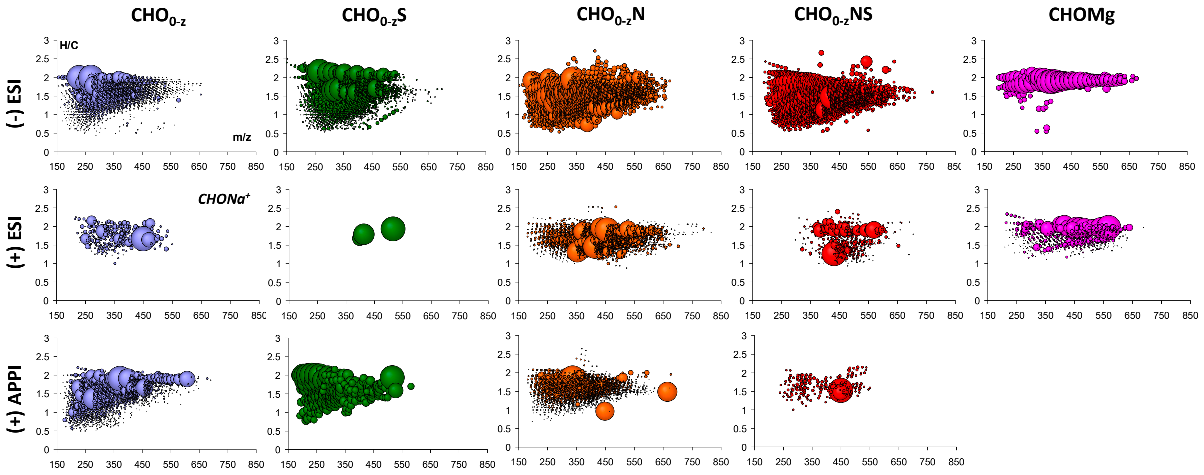

Figure S1. Representation of H/C *vs.* *m*/*z* achieved for the different heteroatom classes identified in the organic extract of Murchison by ESI and APPI FT-ICR MS in positive- and negative-ion modes. The bubble size refers to the corresponding signal intensity.

$C_{16}H_{33}MgO_5^+$

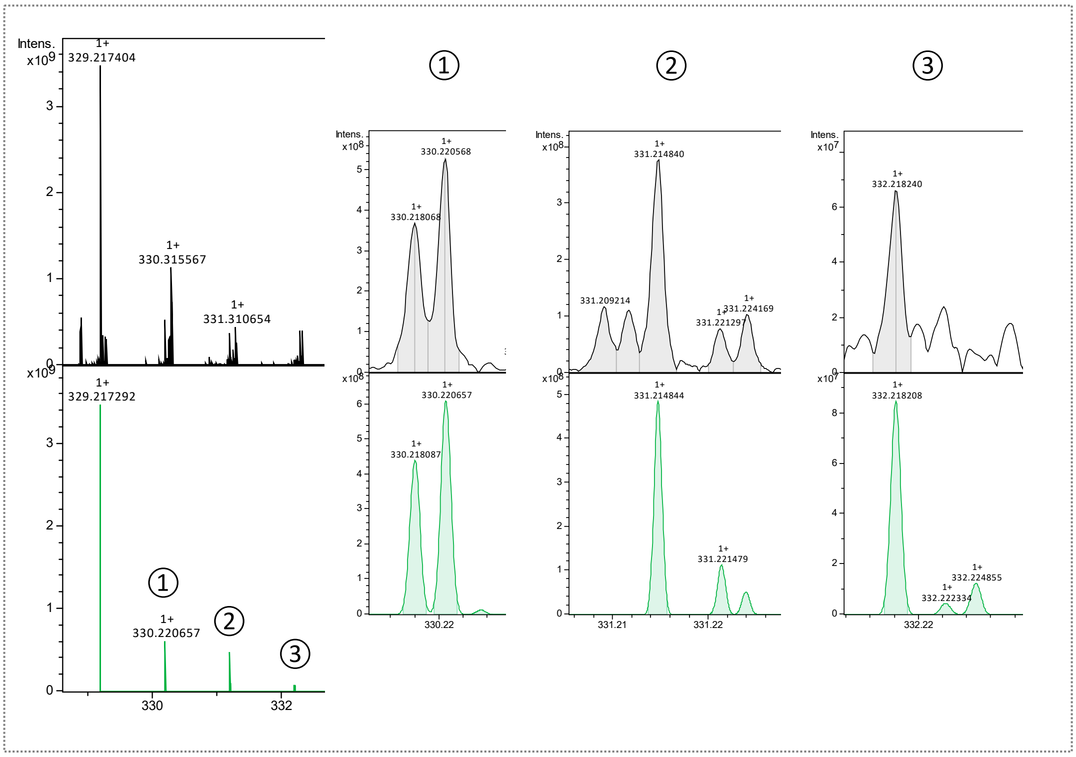

$C_{17}H_{33}MgO_6^+$

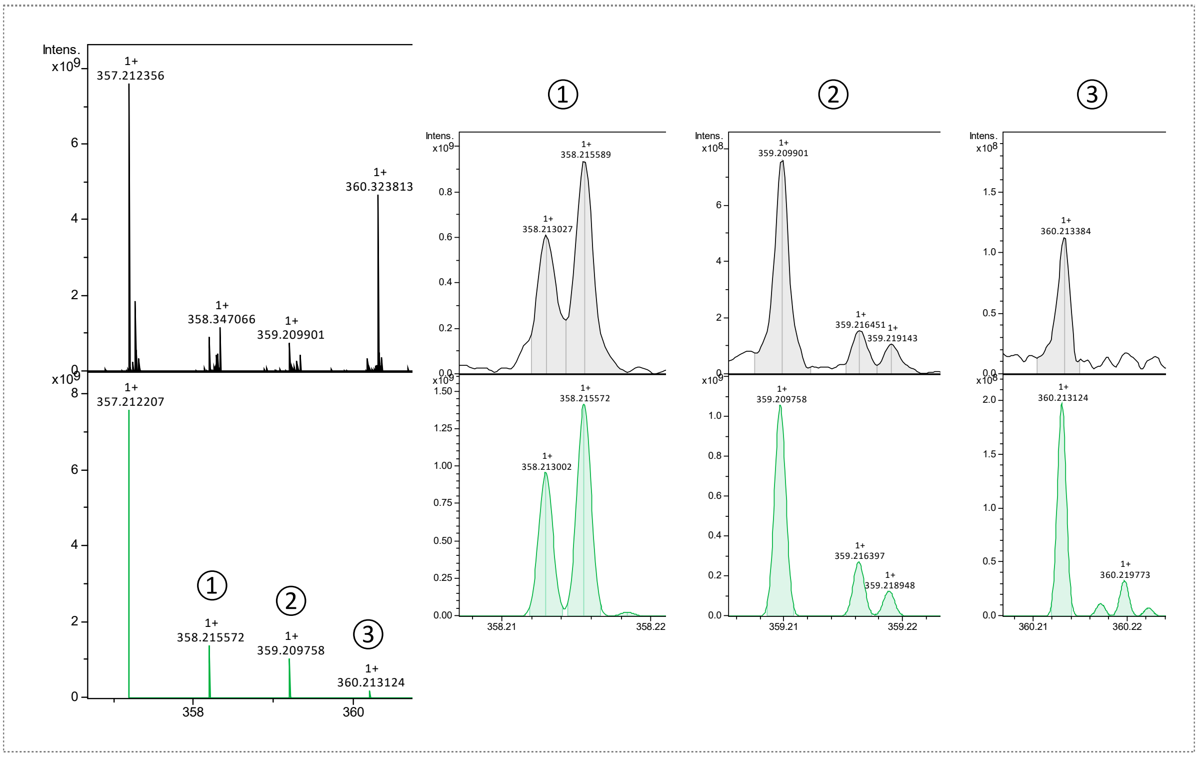

Figure S2. Experimental (grey) and simulated (green) isotopic signals for $[C_{16}H_{33}MgO_5]^+$ (top) and $[C_{17}H_{33}MgO_6]^+$ (bottom) ions identified in the organic extract of Murchison by (+) ESI FT-ICR MS.

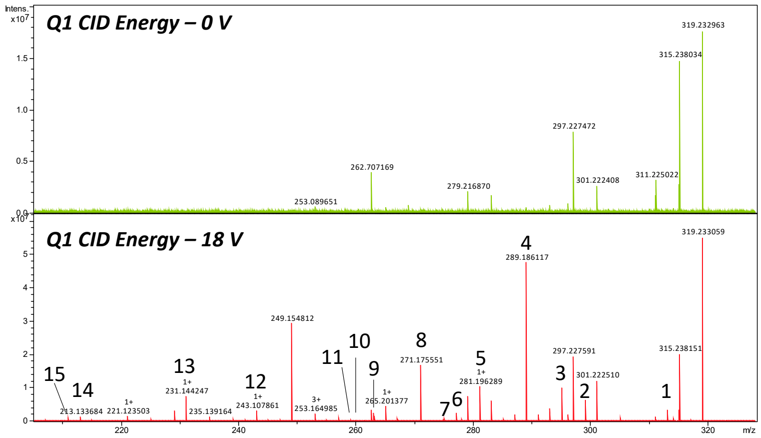

Index	Measured *m/z*	Assignment	Mass error (ppm)
	329.217405	$C_{16}H_{33}MgO_5^+$	-0.341
1	313.217405	$C_{16}H_{33}MgO_4^+$	-0.357
2	299.206853	$C_{15}H_{31}MgO_4^+$	-0.419
3	295.211941	$C_{16}H_{31}MgO_3^+$	-0.433
4	289.186117	$C_{13}H_{29}MgO_5^+$	-0.432
5	281.196289	$C_{15}H_{29}MgO_3^+$	-0.505
6	277.201388	$C_{16}H_{29}MgO_2^+$	-0.447
7	275.185728	$C_{16}H_{27}MgO_2^+$	-0.471
8	271.175551	$C_{13}H_{27}MgO_4^+$	-0.455
9	263.185723	$C_{15}H_{27}MgO_2^+$	-0.476
10	259.175567	$C_{12}H_{27}MgO_4^+$	-0.537
11	257.123539	$C_{11}H_{21}MgO_5^+$	-0.574
12	243.107861	$C_{10}H_{19}MgO_5^+$	-0.491
13	231.144247	$C_{10}H_{23}MgO_4^+$	-0.519
14	213.133684	$C_{10}H_{21}MgO_3^+$	-0.569
15	211.118036	$C_{10}H_{19}MgO_3^+$	-0.586

Figure S3. Fragmentation experiments performed to assess the structure of the organomagnesium component $[C_{16}H_{33}MgO_5]^+$ @ *m/z* 329.217405 in (+) ESI FT-ICR MS. On the top, mass spectrum acquired without collision energy. Below, mass spectrum acquired after isolation and fragmentation of the parent ion. Both mass spectra were acquired after 10 scans accumulation. The Table on the right gathers the different assignments obtained after fragmentation of the $[C_{16}H_{33}MgO_5]^+$ ion.

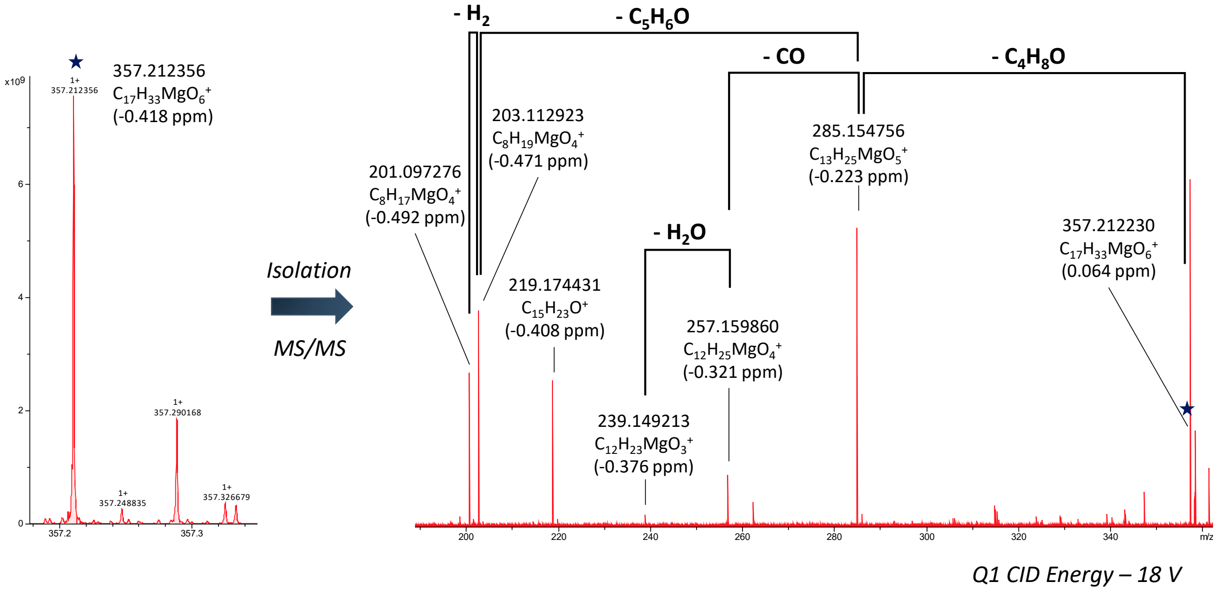

Figure S4. Fragmentation experiments performed to assess the structure of the organomagnesium component $[C_{17}H_{33}MgO_6]^+$ @ *m/z* 357.212230 in (+) ESI FT-ICR MS. The expansion of the fragmentation mass spectrum, on the right, shows the loss of different organic moieties.

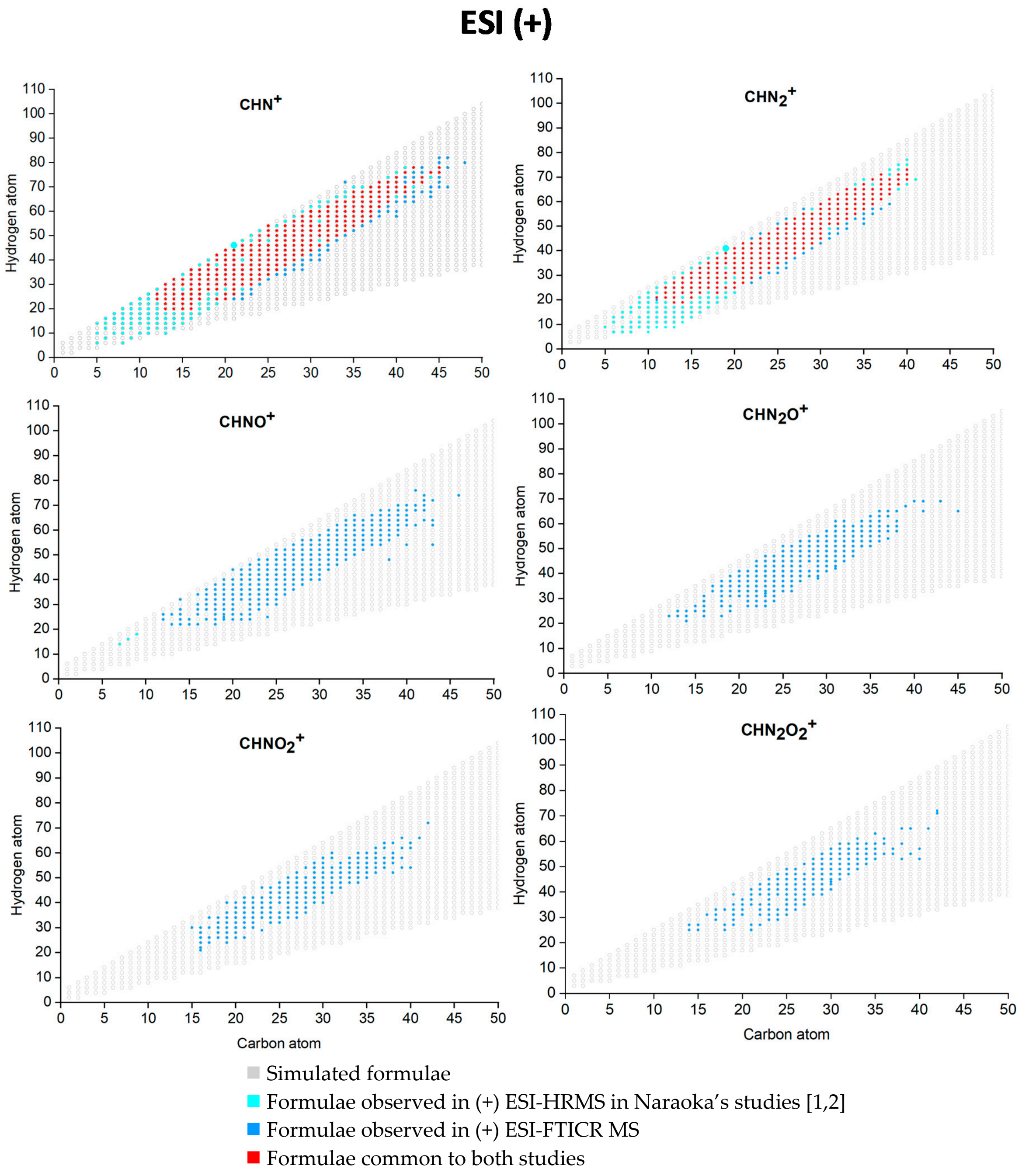

Figure S5. Simulated and experimental $CHN_{1-2}O_{1-2}^{+}$ formulae plotted according to their hydrogen and carbon atoms. The experimental formulae were achieved in this study in positive-ion ESI FT-ICR MS and by Naraoka et al. [1,2].

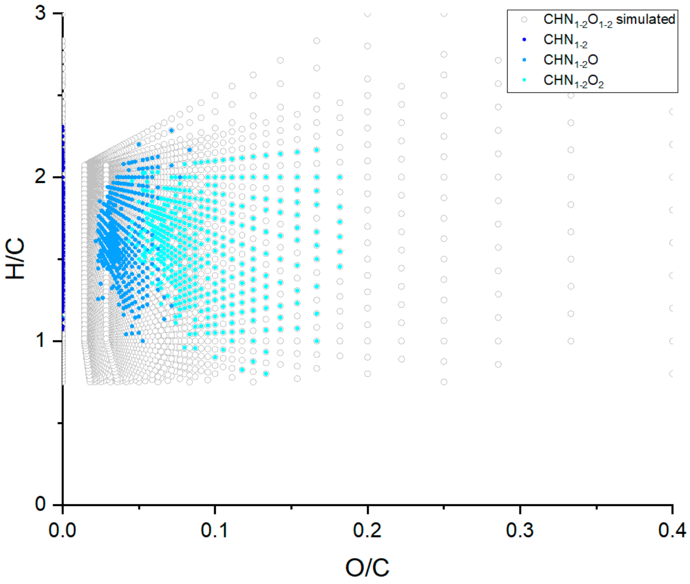

Figure S6. Simulated and experimental $CHN_{1–2}O_{1–2}^{+}$ formulae plotted on van Krevelen diagram. The experimental formulae were achieved in this study in positive-ion ESI FT-ICR MS.

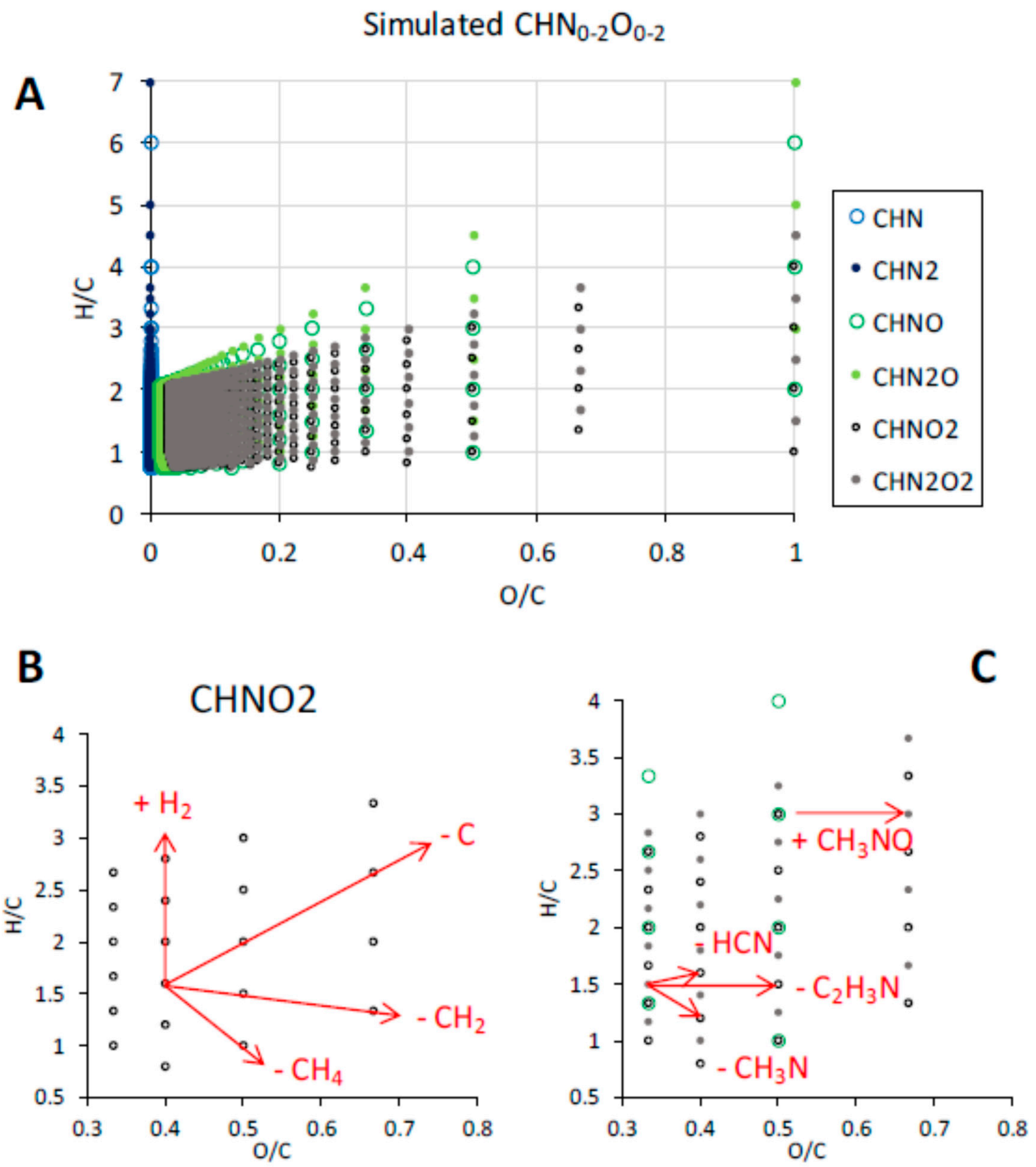

Figure S7. Simulated $CHN_{1–2}O_{1–2}^{+}$ formulae plotted on van Krevelen diagram (**A**). Expansions (**B** and **C**) have been done to observe the different chemical reactions within a same compound class (**B**) or between different classes of components (**C**).

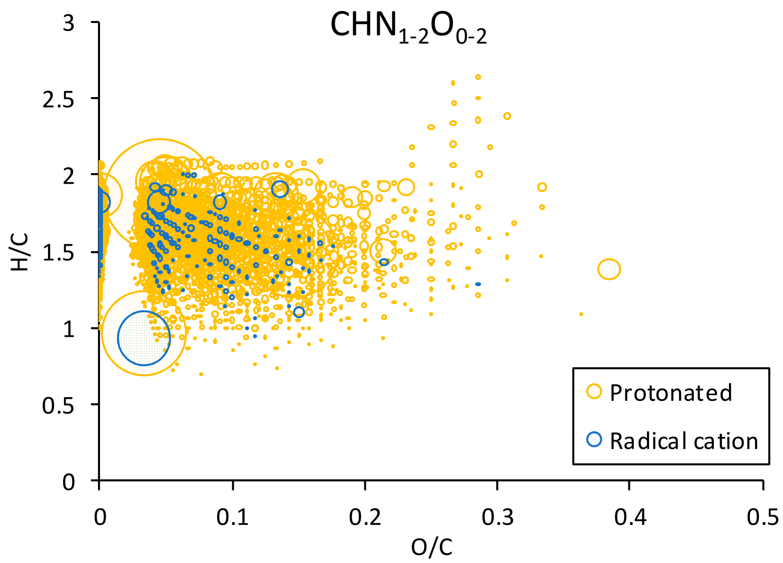

Figure S8. van Krevelen diagram of the CHN and CHNO species assigned in the organic extract of Murchison analysed by (+) APPI FR-ICR MS according to the detection form (protonated or radical). Bubble size is relative to signal intensity of the mass spectrum.

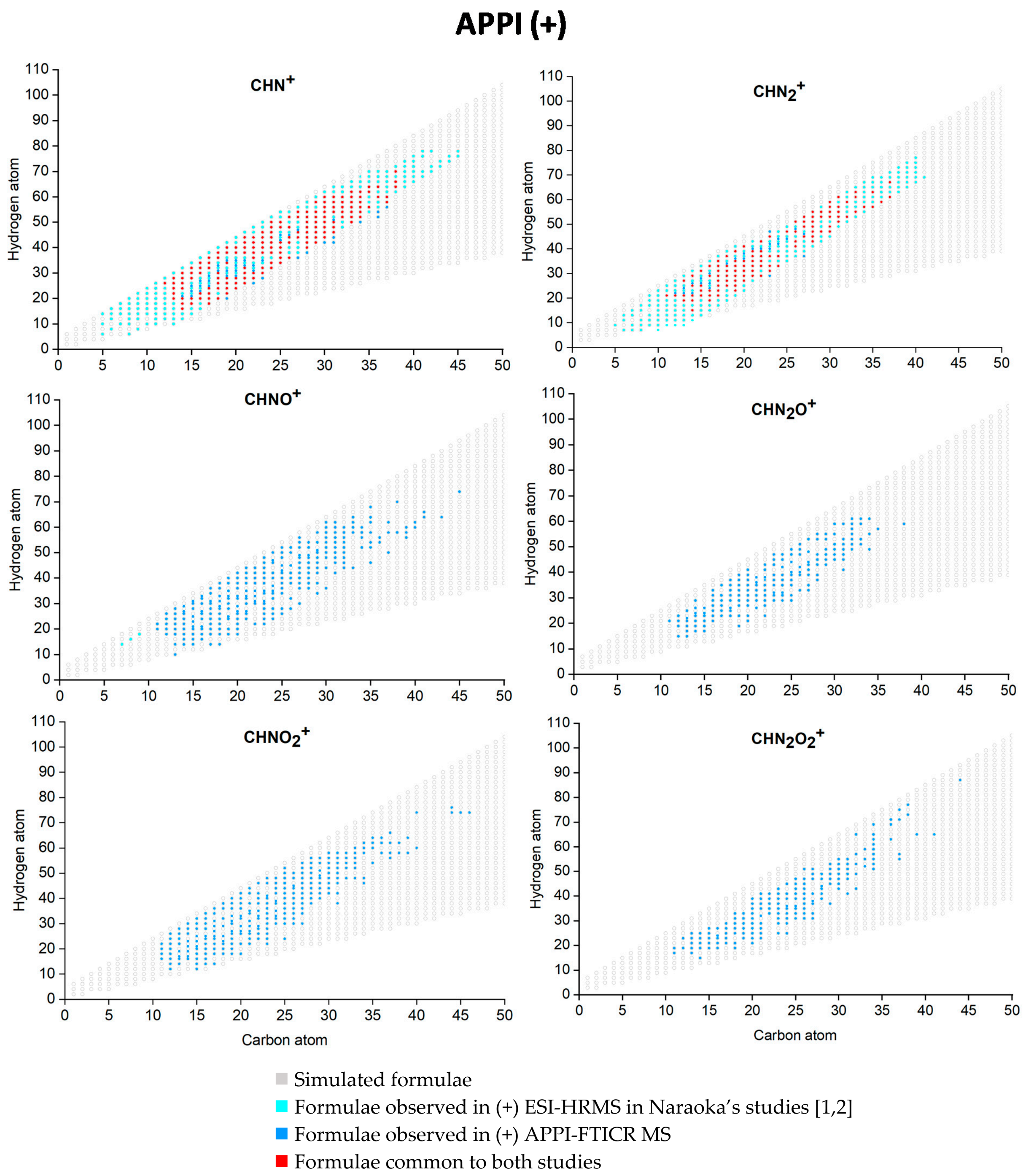

Figure S9. Simulated and experimental $CHN_{1-2}O_{1-2}^{+}$ formulae plotted according to their hydrogen and carbon atoms. The experimental formulae were achieved in this study in positive-ion APPI FT-ICR MS and by Naraoka et al. [1,2].

- χ_c calculated with *m*= 0.5

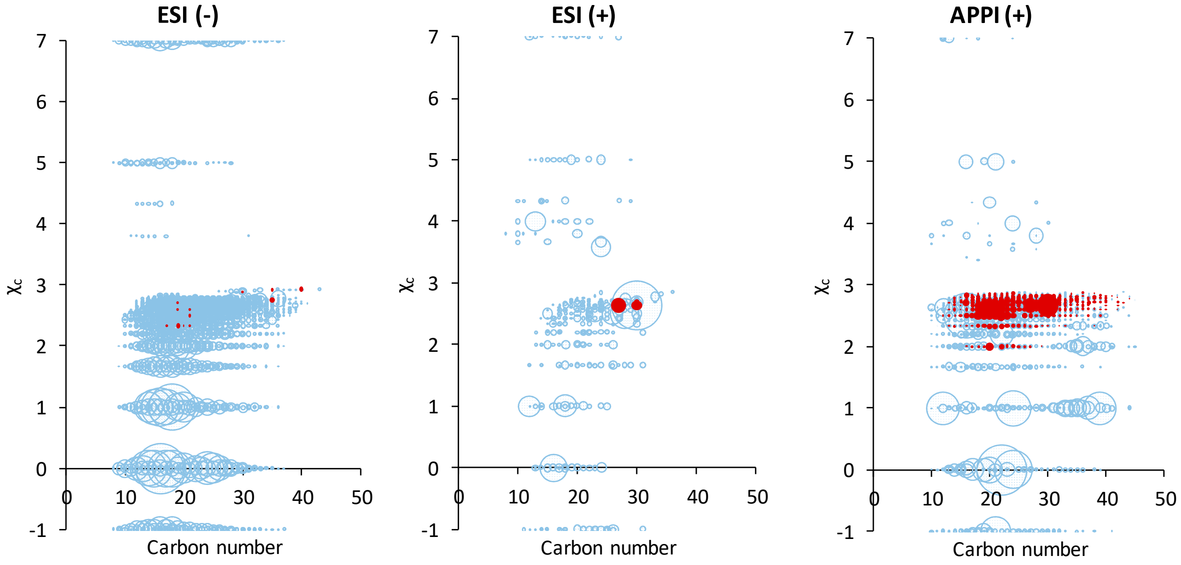

- χ_c calculated with *m*= 1

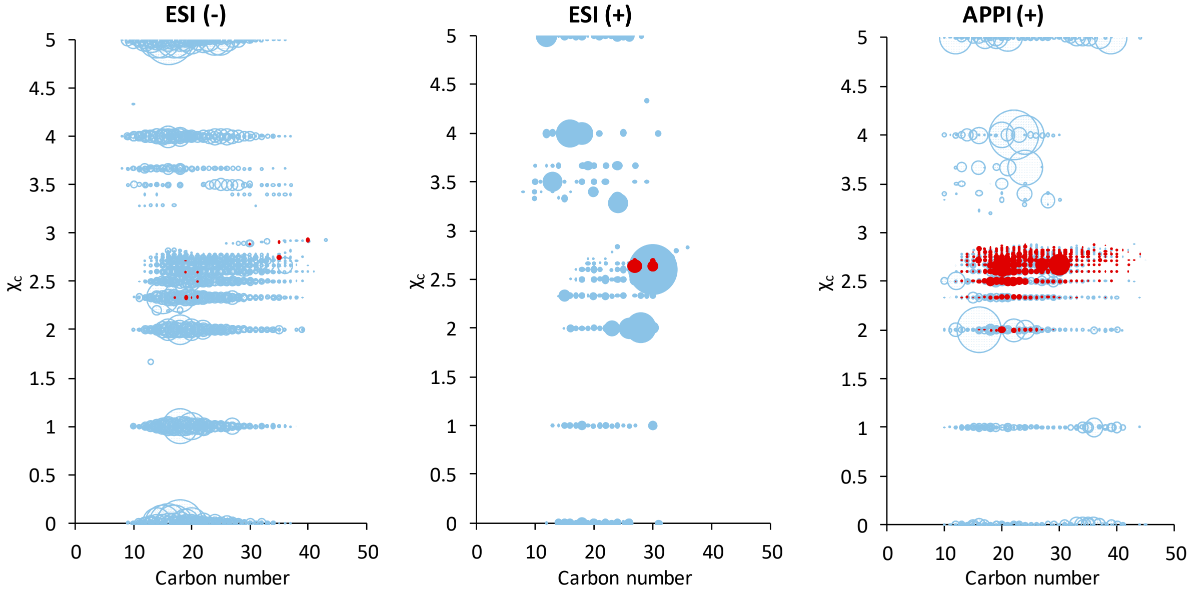

Figure S10. Aromaticity equivalent (χ_c) calculated with m = 0.5 and 1, for CH and CHO assignments obtained by ESI and APPI FT-ICR MS in both detection modes.

References

1. Naraoka, H.; Yamashita, Y.; Yamaguchi, M.; Orthous-Daunay, F.-R. Molecular Evolution of N-Containing Cyclic Compounds in the Parent Body of the Murchison Meteorite. *ACS Earth Space Chem.* **2017**, *1*, 540–550.
2. Hashiguchi, M.; Naraoka, H. High-Mass Resolution Molecular Imaging of Organic Compounds on the Surface of Murchison Meteorite. *Meteorit. Planet. Sci.* **2019**, *54*, 452–468, doi:10.1111/maps.13211.
